# Metabolomics profiles delineate uridine deficiency contributes to mitochondria-mediated apoptosis induced by celastrol in human acute promyelocytic leukemia cells

**DOI:** 10.18632/oncotarget.10286

**Published:** 2016-06-25

**Authors:** Xiaoling Zhang, Jing Yang, Minjian Chen, Lei Li, Fei Huan, Aiping Li, Yanqing Liu, Yankai Xia, Jin-ao Duan, Shiping Ma

**Affiliations:** ^1^ Department of Pharmacology of Chinese Materia Medica, China Pharmaceutical University, Nanjing 210009, China; ^2^ Department of Hygienic Analysis and Detection, Nanjing Medical University, Nanjing 211166, China; ^3^ State Key Laboratory of Reproductive Medicine, Institute of Toxicology, Nanjing Medical University, Nanjing 211166, China; ^4^ Key Laboratory of Modern Toxicology of Ministry of Education, Nanjing Medical University, Nanjing 211166, China; ^5^ Safety Assessment and Research Center for Drug, Pesticide and Veterinary Drug of Jiangsu Province, Nanjing Medical University, Nanjing 211166, China; ^6^ National and Local Collaborative Engineering Center of Chinese Medicinal Resources Industrialization and Formulae Innovative Medicine, Nanjing University of Chinese Medicine, Nanjing 210023, China

**Keywords:** celastrol, acute promyelocytic leukemia, apoptosis, metabolomics, uridine

## Abstract

Celastrol, extracted from “Thunder of God Vine”, is a promising anti-cancer natural product. However, its effect on acute promyelocytic leukemia (APL) and underlying molecular mechanism are poorly understood. The purpose of this study was to explore its effect on APL and underlying mechanism based on metabolomics. Firstly, multiple assays indicated that celastrol could induce apoptosis of APL cells via p53-activated mitochondrial pathway. Secondly, unbiased metabolomics revealed that uridine was the most notable changed metabolite. Further study verified that uridine could reverse the apoptosis induced by celastrol. The decreased uridine was caused by suppressing the expression of gene encoding Dihydroorotate dehydrogenase, whose inhibitor could also induce apoptosis of APL cells. At last, mouse model confirmed that celastrol inhibited tumor growth through enhanced apoptosis. Celastrol could also decrease uridine and DHODH protein level in tumor tissues. Our *in vivo* study also indicated that celastrol had no systemic toxicity at pharmacological dose (2 mg/kg, i.p., 21 days). Altogether, our metabolomics study firstly reveals that uridine deficiency contributes to mitochondrial apoptosis induced by celastrol in APL cells. Celastrol shows great potential for the treatment of APL.

## INTRODUCTION

Acute promyelocytic leukemia (APL), characterized by a differentiation block at the promyelocytic stage, is one of the most dangerous leukemias and the most frequent cause of leukemia-related deaths in the United States [1]. Despite recent advances in the treatment of this disease with the application of all-*trans* retinoic acid (ATRA) and arsenic trioxide (ATO), treatment failure still often occurs [2, 3]. In addition, ATRA and ATO are both water-soluble chemicals. They have limited ability to cross the blood-brain barrier, and cannot reach therapeutically effective levels in the cerbrospinal fluid. Therefore, they are helpless for extramedullary relapse, which happens most commonly in the central nervous system in APL patients [4].

So it is urgent to find new drugs with higher efficacy and lower toxicity for the treatment of APL. Fortunately, bioactive natural products open new avenues for us [5–7]. Recently, light has been shed on the active constituent from traditional Chinese medicine. The discoverer of artemisinin, Chinese pharmacologist Youyou Tu, was awarded the 2015 Nobel Prize in Physiology or Medicine.

Celastrol, a pentacyclic triterpene isolated from the roots of “Thunder of God Vine”, has aroused extensive attention due to its potential in the treatment of inflammatory and auto-immune diseases [8] and obesity [9, 10]. Recently, its anti-cancer [11–14] and chemotherapy sensitization activities [15–17] were increasingly focused. However, the anti-leukemia effect of celastrol is rarely reported, especially on APL. HL-60 is a widely used cell line for the study of APL. Although there are two reports indicating that celastrol could trigger apoptosis of HL-60 cells [18, 19], the underlying molecular mechanism is poorly understood.

Metabolic disturbance is the hallmark of cancer cell. Metabolomics, an emerging omics technology, is an ideal tool to monitor the metabolic alterations, which is increasingly being used for the supervision of pathophysiologic processes of diseases as well as toxicity and pharmacological assessment of chemical exposure [20–22]. So we chose metabolomics as a sally port to investigate the mechanism underlying the effect of celastrol on APL cells.

Here, for the first time, by conducting a hypothesis free metabolomics analysis, we find that uridine deficiency contributes to mitochondrial apoptosis induced by celastrol in human APL cells. Celastrol shows great potential for the treatment of APL.

## RESULTS

### Celastrol induces apoptosis of HL-60 cells

**Figure 1:**
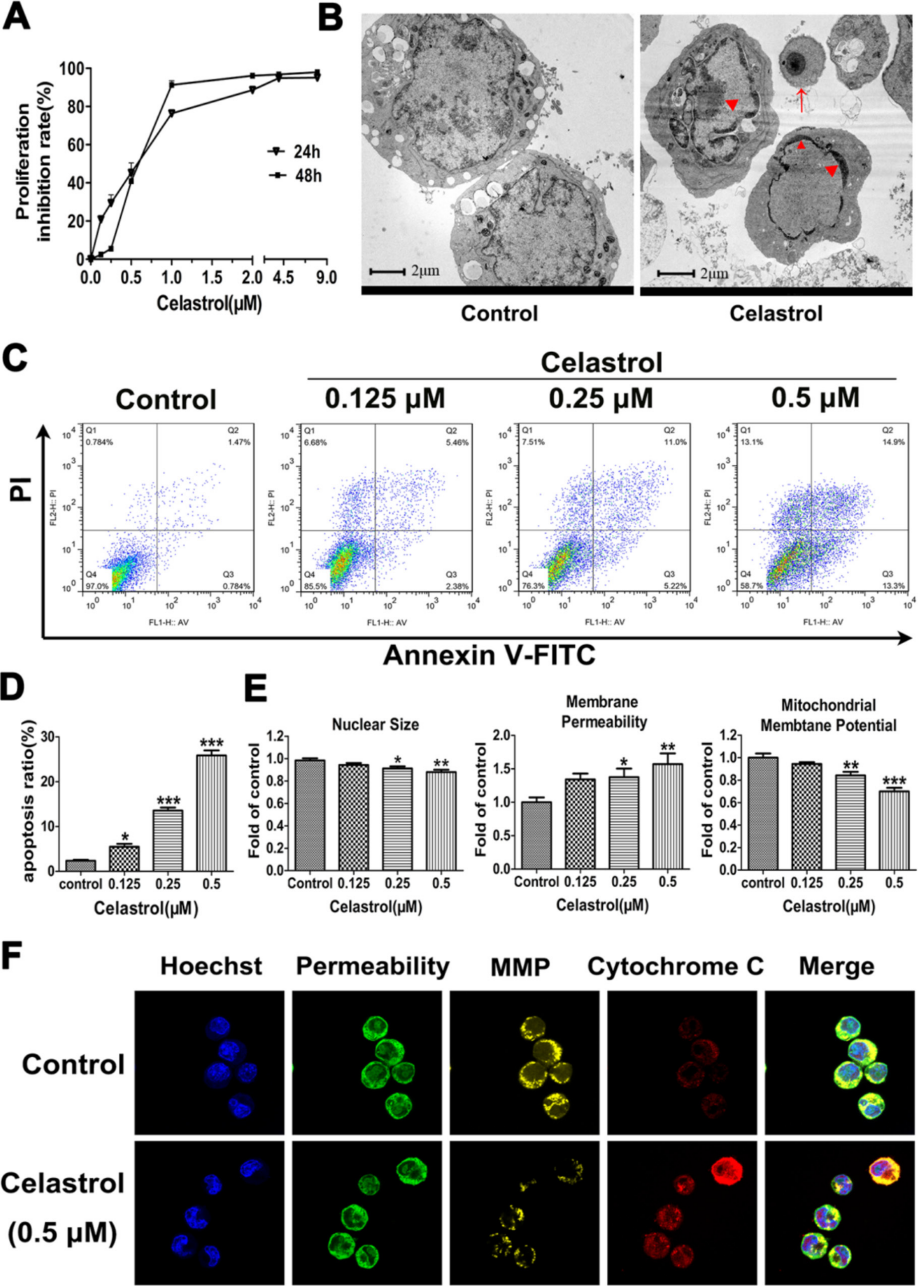
The effects of celastrol on cell proliferation and apoptosis in HL-60 cells **A.** Effect of celastrol on cell proliferation. Data represented the mean of five replicates. Each performed in triplicate. **B.** Characteristic apoptotic morphological changes were assessed by transmission electron microscopy. The concentration of celastrol was 0.5 μM. Arrow indicates apoptotic body and triangles indicate nuclear condensation and margination. Scale bar: 2 μm; Magnification, ×20,000. **C.** Flow cytometry analysis of cell apoptosis. **D.** The chart illustrates the percentage of apoptotic cells from three independent experiments. **E.** The quantitative analysis of nuclear size, membrane permeability and mitochondria membrane potential. **F.** Representative images from confocal microscope. MMP: Mitochondrial Membrane Potential. The concentration of celastrol was 0.5 μM. Magnification, ×630. Asterisks indicate statistical significance (*p < 0.05, **p < 0.01, ***p < 0.001).

In order to evaluate the effect of celastrol on cell proliferation, the CCK-8 assay was performed. As shown in Figure 1A, a dose-dependent cell proliferation inhibition on HL-60 cells was observed after treatment with celastrol for 24 and 48 h. The concentration of celastrol to reach 50% proliferation inhibition (IC_50_) was 0.48 and 0.55 μM for 24 and 48 h, respectively. There was no significant difference between 24 and 48 h treatment. According to the IC_50_ value and previous report [23], we selected 0.125, 0.25 and 0.5 μM as the test doses and 24 h as the treatment time in the following study regarding the effect of celastrol on HL-60 cells.

The cell ultra-structure changes caused by celastrol were then assessed by transmission electron microscope. As shown in Figure 1B, cell shrinkage, chromatin margination and condensation, smooth cell membrane and formation of apoptotic bodies were observed in HL-60 cells treated by celastrol. Noticeably, these changes were all characteristics of cells undergoing apoptosis. Therefore, the apoptosis was next quantified by flow cytometry with Annexin V/PI staining. As shown in Figure 1C and 1D, the percentage of apoptotic cells induced by celastrol was significantly increased in a dose-dependent manner, indicating that celastrol has potent pro-apoptotic effect on HL-60 cells.

### The pro-apoptotic effect of celastrol is executed through p53-activated mitochondrial pathway

In order to explore the mechanisms underlying celastrol-induced apoptosis of HL-60 cells, the effects of celastrol on nuclear morphology, cell membrane permeability and mitochondrial membrane potential changes were evaluated using High Content Screening multi-parameter cytotoxicity analysis. As shown in Figure 1E and Supplementary Figure S1, quantitative analysis indicated that nuclear size became smaller, and cell membrane permeability was increased, and mitochondrial membrane potential was decreased in a dose-dependent manner after celastrol treatment. Meanwhile, these results were verified by confocal microscopy analysis (Figure 1F and Supplemenatary Figure S2). As shown in Figure 1F, the cytochrome c mainly located in the mitochondria in control group cells. While in the celastrol-treated cells, some cytochrome c was found outside the mitochondria, indicating the increased releasing of cytochrome c from mitochondria to cytoplasm. Collectively, celastrol-triggered apoptosis may be executed through mitochondrial pathway in HL-60 cells.

**Figure 2:**
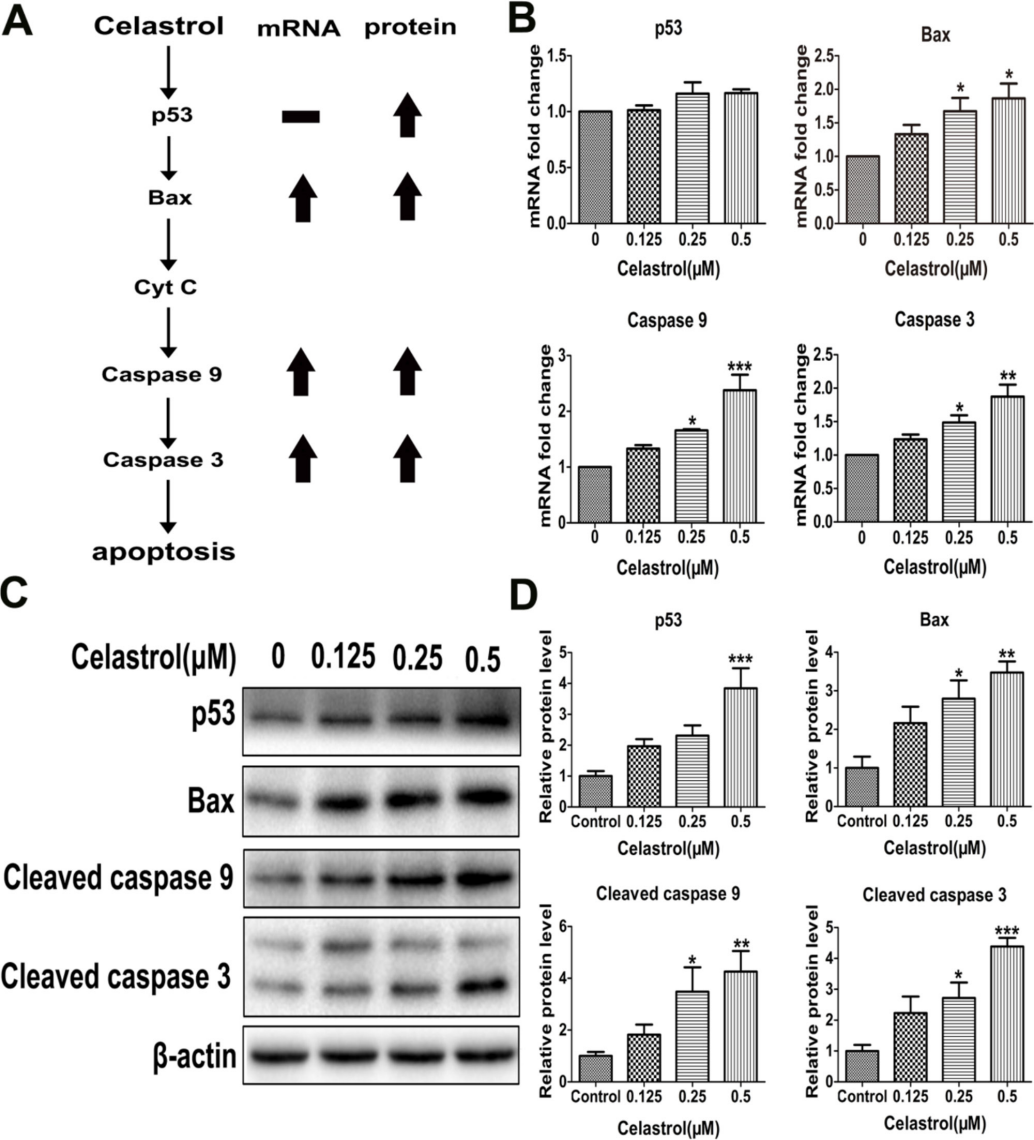
The mRNA and protein levels of apoptosis-related genes **A.** Schematic illustration of apoptosis pathway. **B.** Real-time PCR analysis of *TP53*, *BAX*, *CASPASE 9* and *CASPASE 3* mRNA levels. **C.** The protein levels of cleaved caspase 9, cleaved caspase 3, p53 and Bax with western blot analysis. **D.** The integrated option density of target protein bands was quantified by Image-Pro Plus software. Results represent three independent experiments performed in triplicate. Asterisks indicate statistical significance (*p < 0.05, **p < 0.01, ***p < 0.001).

On one hand, the released cytochrome c may activate caspase 9, which activates caspase 3 subsequently. On the other hand, the release of cytochrome c is controlled by Bcl-2 family proteins, especially Bax. Besides, the expression of Bax can be activated by tumor suppressor p53 [24] (Figure 2A). Therefore, we detected the expression of caspase 9, caspase 3, Bax and p53. After celastrol treatment, except p53, the mRNA levels of caspase 9, caspase 3 and Bax were increased (Figure 2B), and the protein levels of cleaved caspase 9, cleaved caspase 3, Bax and p53 were all increased significantly (Figure 2C and 2D). Additionally, the expression of caspase 8, an important participator of extrinsic apoptosis pathway was also detected. The mRNA level of caspase 8 was decreased significantly (Supplementary Figure S3). Taken together, all the results demonstrated that the apoptosis of HL-60 cells induced by celastrol was through p53-activated mitochondrial pathway but not death receptor pathway. Furthermore, in order to explore the upstream of mitochondrial apoptosis pathway and find new targets of celastrol action, we conducted an unbiased metabolomics analysis.

### Metabolomics reveals that uridine is the most notable changed metabolite influenced by celastrol

**Figure 3:**
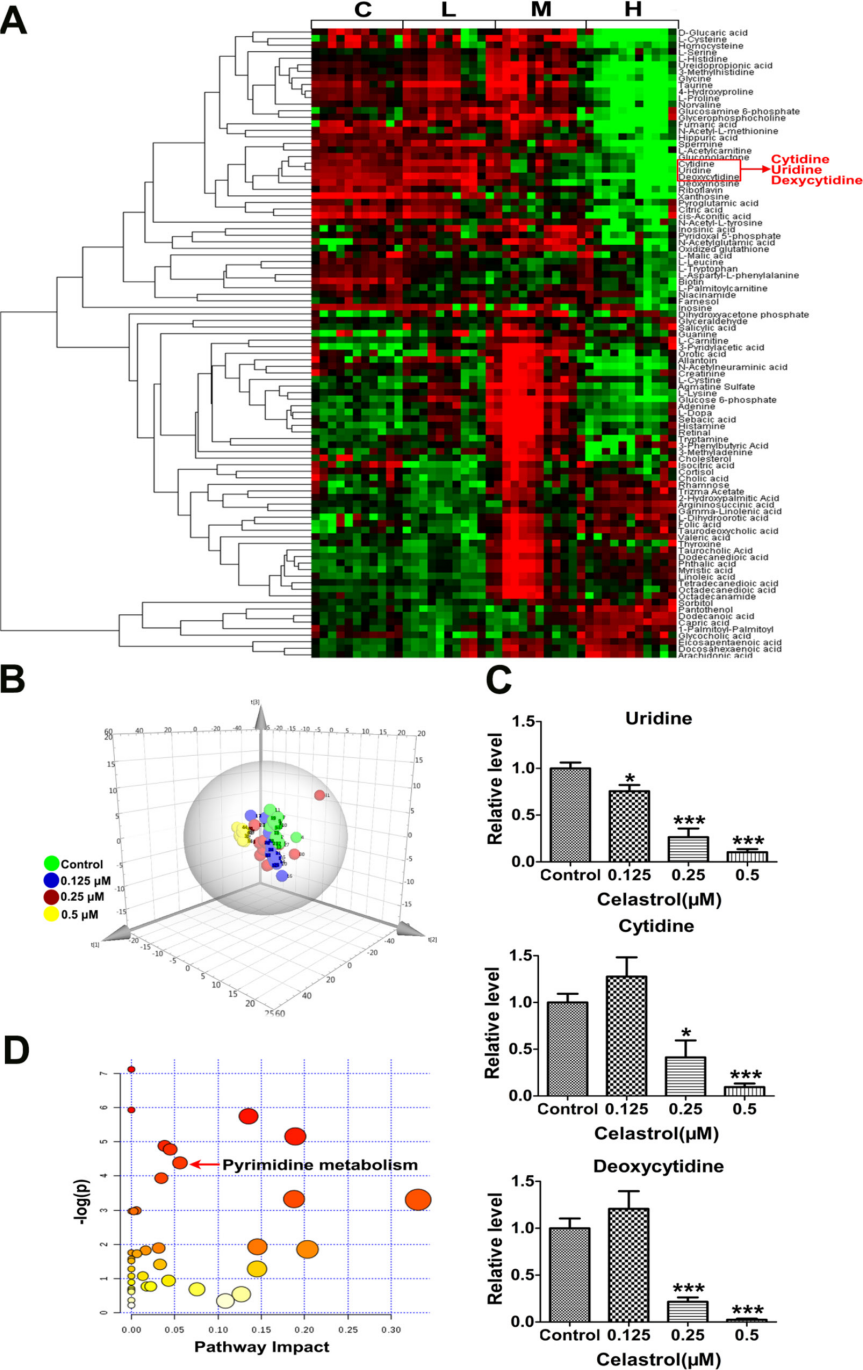
Metabolomics profiles **A.** Heat map showing 95 differential metabolites. C: control, L: 0.125 μM celastrol, M: 0.25 μM celastrol, H: 0.5 μM celastrol. **B.** PCA score plot derived from metabolomics analysis. Green circle indicated the control group. Blue circle indicated the 0.125 μM celastrol treatment. Red circle indicated the 0.25 μM celastrol treatment. Yellow circle indicated the 0.5 μM celastrol treatment. **C.** The relative content of uridine, cytidine and deoxycytidine in HL-60 cells with or without celastrol treatment. Asterisks indicate statistical significance (*p < 0.05, ***p < 0.001). **D.** The pathway analysis of metabolomics data.

In metabolomics analysis, a total of 192 metabolites were detected. After excluding metabolites with a detection rate lower than 50%, we retained 95 metabolites for further analysis. The profiles are displayed as a heat map (Figure 3A).

Principal Components Analysis (PCA) is an unsupervised, reductive statistical modeling technique which separates samples based on their differences from each other. As shown in Figure 3B, the established PCA model showed good discrimination for celastrol (0.125 μM), celastrol (0.25 μM), celastrol (0.5 μM) and control group in a dose-response manner, suggesting celastrol treatment had a dose-related influence on metabolism of HL-60 cells. So we focused on the effect of high-dose (0.5 μM) celastrol treatment. We found 67 metabolites were significantly changed in 0.5 μM celastrol treatment group (Supplementary Table S1, p < 0.05). Meanwhile, in order to improve the robustness of the result, we applied a Bonferroni correction for multiple testing based on an adjusted p-value threshold of 5×10^−4^ according to previous study [25]. Finally, 39 metabolites were retained (Supplementary Table S1). After mapping these metabolites into their respective biochemical pathways as outlined in the Kyoto Encyclopedia of Genes and Genomes (KEGG) (http://www.genome.jp/kegg/), we found that three of the top seven notable metabolites were within pyrimidine metabolism pathway (Supplementary Table S1). The three pyrimidine metabolites were uridine, cytidine and deoxycytidine, and their content changes are shown in Figure 3C. Interestingly, the three metabolites were clustered in the heat map (Figure 3A), highlighting the statistical importance of the three metabolites. We next used the module of “pathway analysis” of metaboanalyst (www.metaboanalyst.ca/) to do the pathway enrichment. We found pyrimidine metabolism was the significantly changed metabolic pathway (Figure 3D and Supplementary Table S2). These results collectively indicated that celastrol had potent effect on the pyrimidine metabolism in HL-60 cells. Furthermore, among the three metabolites within pyrimidine metabolism, uridine was a representative pyrimidine nucleoside which has metabolic connection with cytidine and deoxycytidine and showed the most robust dose-dependent response to celastrol (Figure 3C). Besides, it was reported that uridine deficiency could increase the protein level of p53 by preventing its degradation [26], which can well explain the inconsistence between mRNA and protein level of p53 after celastrol treatment. Therefore, the decreased uridine caused by celastrol may increase p53-activted apoptosis in HL-60 cells, which led us to further validate this hypothesis.

### Uridine reverses apoptosis induced by celastrol in HL-60 cells

To evaluate the role of uridine in apoptosis induced by celastrol, the HL-60 cells were treated with uridine in conjunction with celastrol (0.5 μM). The enriched concentrations of uridine were 25, 50 and 100 μM in the culture medium according to previous studies [27–30].

**Figure 4:**
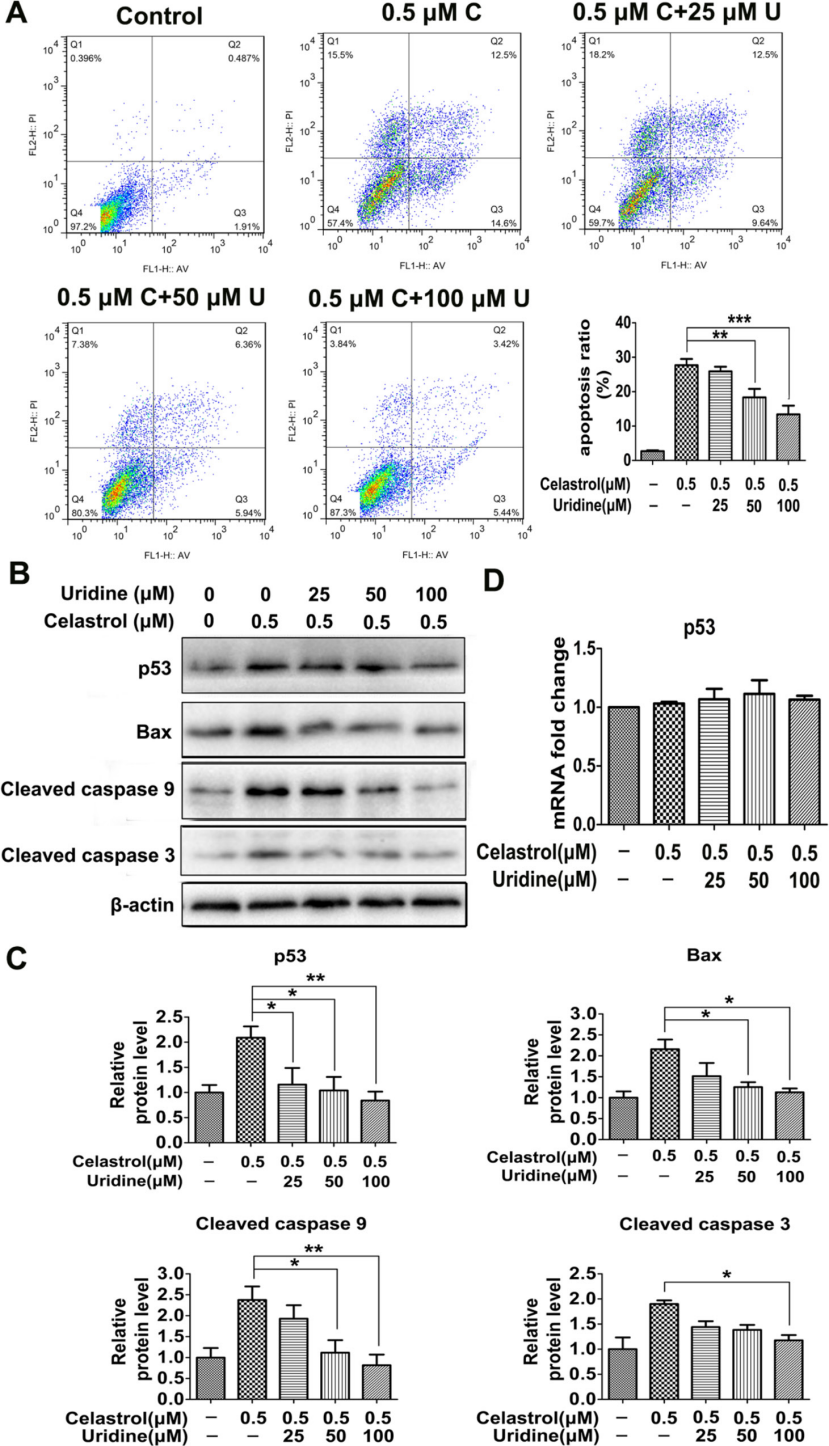
The apoptosis induced by celastrol was attenuated by the addition of uridine **A.** Flow cytometry was conducted to evaluate the effect of uridine supplementation on apoptosis. C: Celastrol; U: uridine. **B.** and **C.** The effect of uridine on the protein levels of apoptosis-related genes. **D.** The effect of celastrol or the combination of celastrol and uridine on the mRNA level of p53. Asterisks indicate statistical significance (*p < 0.05, **p < 0.01, ***p < 0.001).

As shown in Figure 4A, celastrol treatment induced significant apoptosis of HL-60 cells. However, the addition of uridine decreased the apoptotic percentage of HL-60 cells in a dose-dependent manner. As shown in Figure 4B and 4C, the protein levels of p53, Bax, cleaved caspase 9 and cleaved caspase 3 were significantly increased in the celastrol treatment group. However, the levels of these proteins were decreased with the addition of uridine in a dose-dependent manner. As shown in Figure 4D, celastrol alone or in conjunction with uridine didn't show effect on the mRNA levels of p53. These results support our hypothesis that uridine may play a key role in p53-activated apoptosis triggered by celastrol, and are also in accordance with previous study that uridine deficiency activated p53 response by maintaining the stability of p53 protein [26]. These findings drove us to further investigate the mechanism through which celastrol led to uridine deficiency.

### Celastrol suppresses the expression of DHODH, which enhances the salvage biosynthesis using uridine as substrate

**Figure 5:**
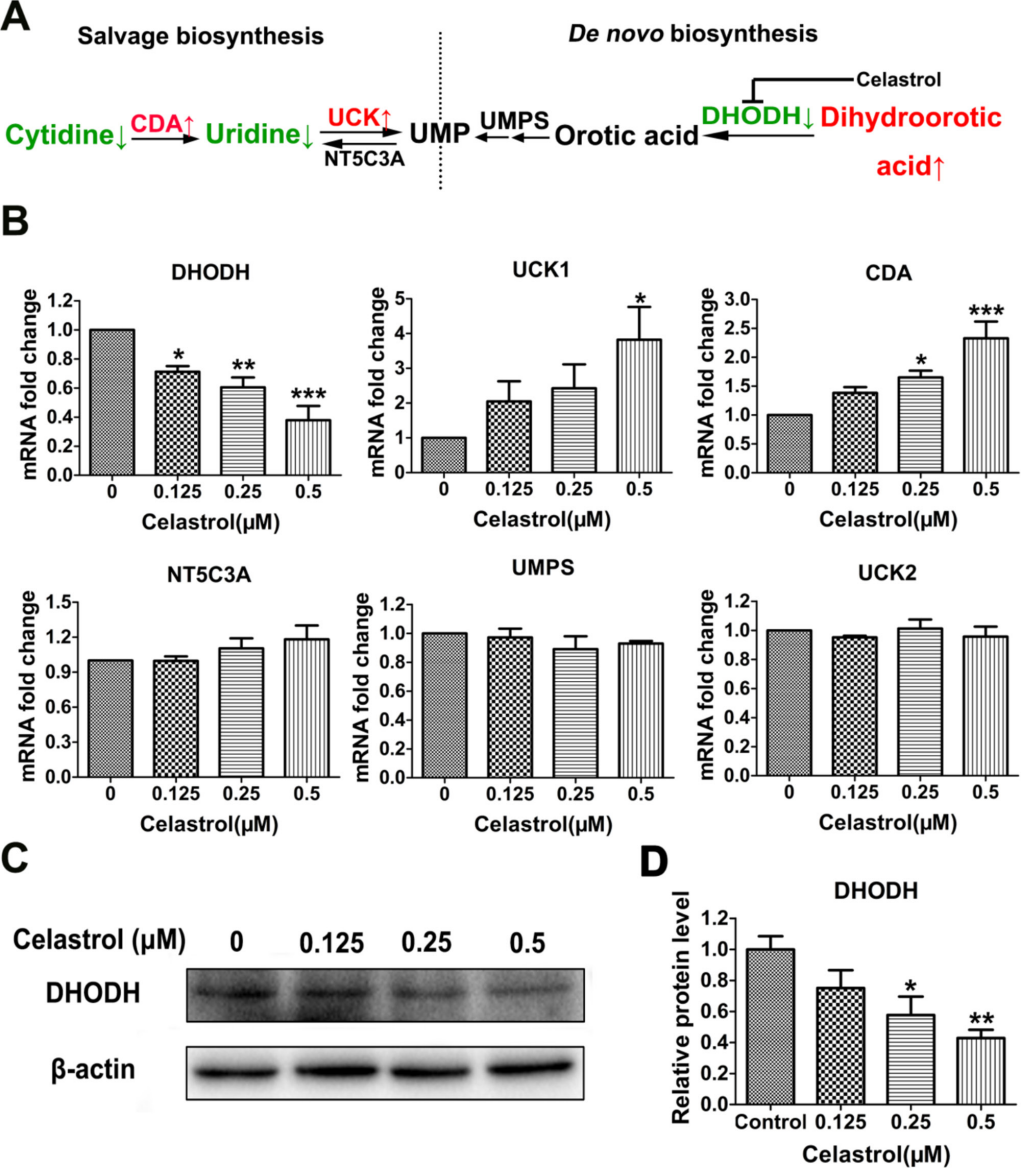
The screening of target metabolic enzyme gene expression affected by celastrol **A.** Schematic overview of pyrimidine biosynthesis. **B.** The mRNA levels of candidate enzyme genes involved in the metabolism of pyrimidine. CDA: cytidine deaminase, EC 3.5.4.5; UCK: uridine-cytidine kinase, EC 2.7.1.48; NT5C3A: 5′-nucleotidase, cytosolic IIIA, EC 3.1.3.5; UMPS: uridine monophosphate synthetase, EC 2.4.2.10 and EC 4.1.1.23;DHODH, dihydroorotate dehydrogenase, EC 1.3.3.1. **C.** Western blot was utilized to validate the protein level of the target enzyme gene. **D.** The integrated option intensity of target protein band was quantified by Image-Pro Plus software. Results represent three independent experiments performed in triplicate. Asterisks indicate statistical significance (*p < 0.05, **p < 0.01, ***p < 0.001).

**Figure 6:**
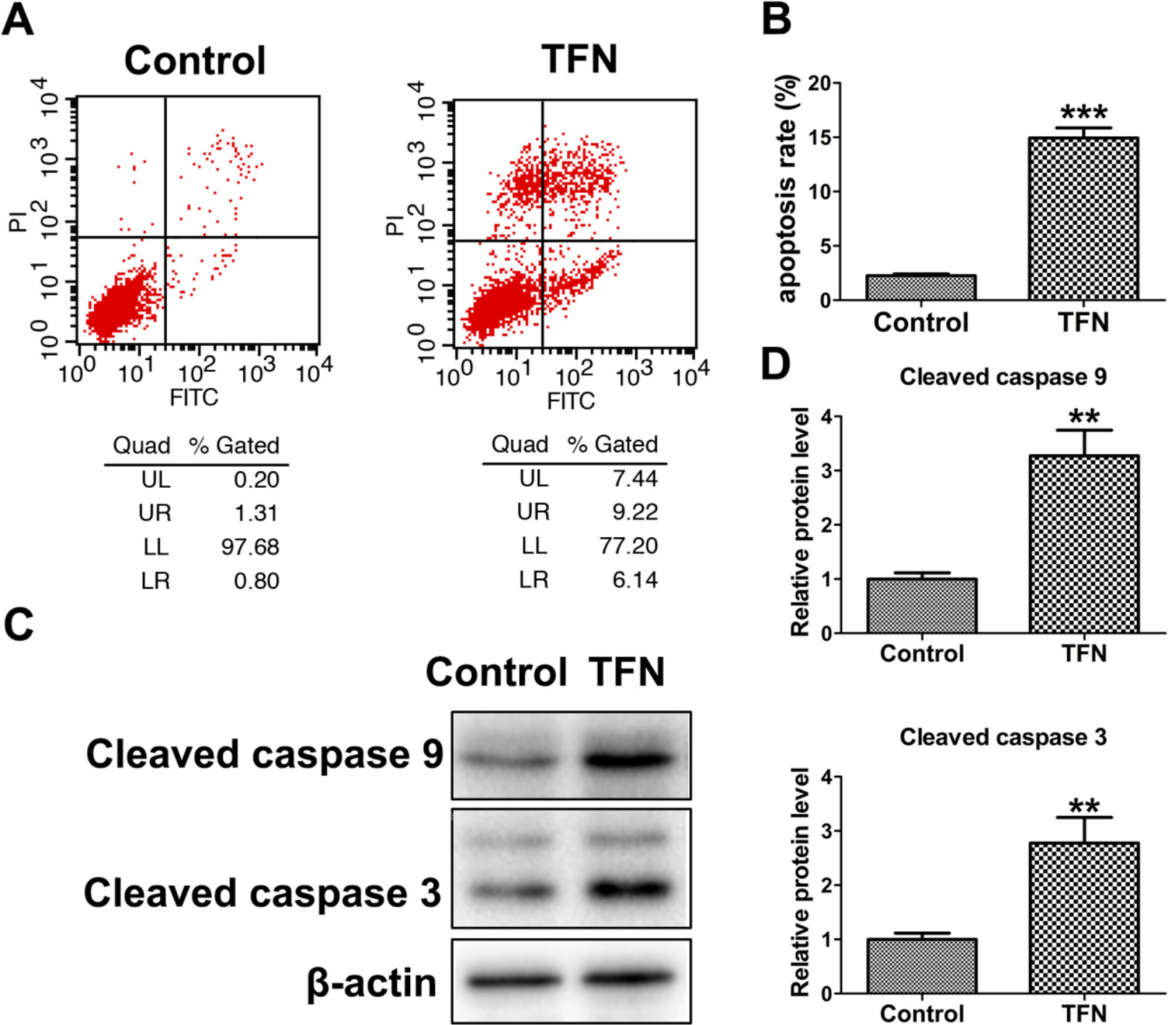
The pro-apoptotic effect of Teriflunomide (TFN, a specific inhibitor of DHODH) on HL-60 cells **A.** and **B.** HL-60 cells were treated with 0.1% DMSO (control) and 200 μM TFN for 24 h. Flow cytometry analysis was performed to determine the apoptosis rate. **C.** and **D.** Western blot analysis showed Teriflunomide (200 μM) increased cleaved caspase 9 and cleaved caspase 3 protein levels in HL-60 cells. Asterisks indicate statistical significance (**p < 0.01, ***p < 0.001).

To identify the target through which celastrol triggered uridine deficiency, we detected the mRNA levels of genes encoding the key enzymes involved in pyrimidine metabolism. The two main pathways for pyrimidine biosynthesis are shown in Figure 5A. Uridine, as a substrate, is mainly used for the salvage biosynthesis of UMP. This reaction is catalyzed by UCK1 and/or UCK2. Meanwhile, CDA is another important enzyme in this pathway, which catalyzes the transformation of cytidine to uridine under the uridine deficiency. As shown in Figure 5B, the mRNA levels of UCK1 and CDA were significantly elevated, while the mRNA level of UCK2 showed no significant changes. The results suggested the enhancement of UMP salvage biosynthesis, which led to uridine deficiency directly. In cancer cells, UMP biosynthesis often mainly depends on *de novo* biosynthesis [28]. The enhancement of salvage biosynthesis may be due to the inhibition of *de novo* biosynthesis. DHODH and UMPS are two important enzymes in this pathway, especially DHODH, which acts as a rate-limiting enzyme. As shown in Figure 5B, the mRNA level of UMPS had no changes, while the mRNA level of DHODH was significantly decreased after celastrol treatment. In addition, the mRNA level of NT5C3A showed no changes. These results suggested that the *de novo* biosynthesis of UMP may be arrested at the DHODH. Interestingly, this was supported by the increase of dihydroorotic acid, the upstream substrate of DHODH (Supplementary Table S1). Additionally, in order to further confirm this hypothesis, we examined the protein level of DHODH. As shown in Figure 5C and 5D, the protein level of DHODH was decreased in a dose-dependent manner. These results indicated celastrol inhibited the *de novo* biosynthesis of UMP by down-regulating the expression of DHODH, which compensatorily enhanced salvage biosynthesis and significantly increased the consumption of uridine as substrate. Moreover, we found that Teriflunomide, a specific inhibitor of DHODH, could also induce apoptosis of HL-60 cells (Figure 6). This result further supported DHODH was the target through which celastrol induced apoptosis of HL-60 cells.

### Celastrol (2 mg/kg/day, for 21 days) has no systemic toxicity in the tumor xenograft nude mice

**Figure 7:**
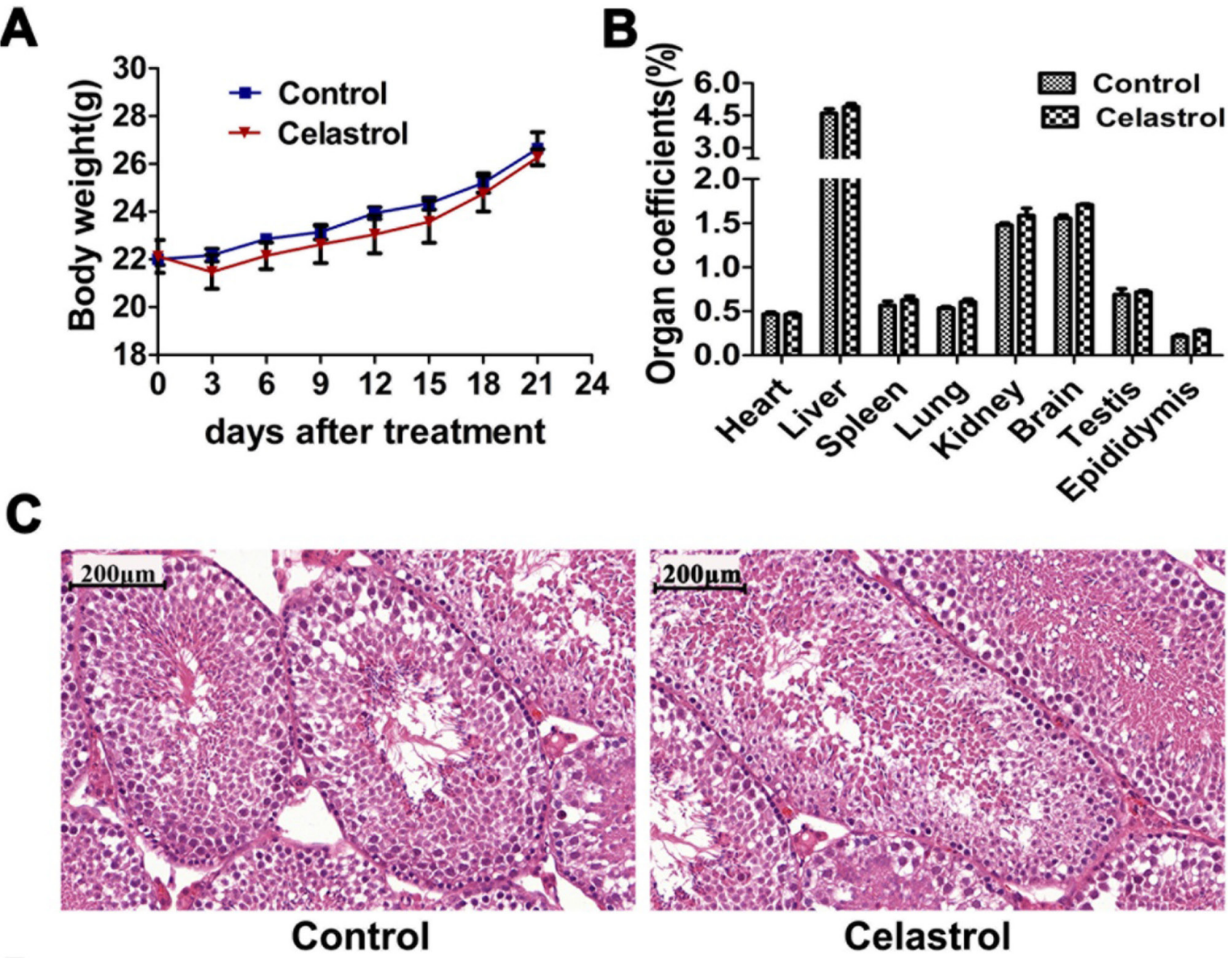
*In vivo* toxicity evaluation of celastrol **A.** Body weight changes of nude mice after celastrol treatment. **B.** Organ coefficients of major organs including heart, liver, spleen, lung, kidney, brain, testis and epididymis. **C.** Optical microscopy images of testis H&E staining after celastrol treatment. Scale bar: 200 μm. Magnification, ×10.

To explore the systemic toxicity of celastrol treatment and validate the findings *in vitro*, we established a model of tumor xenograft nude mice of APL as described previously [31–33]. As shown in Figure 7A, 7B and Supplementary Figure S4, no significant differences between control and celastrol treated mice were observed in body weight and organ coefficients of heart, liver, spleen, lung, kidneys, brain, testis and epididymis. As shown in Table 1, ALT, AST, BUN and CREA showed no statistical differences between control and celastrol-treated mice, and all values were within the normal range [34], indicating celastrol had no toxicity on liver and kidney at dose of 2 mg/kg. In addition, no noticeable histopathological changes were observed in testis of mice treated with celastrol (Figure 7C), indicating celastrol had no toxicity on reproduction system.

**Table 1:** Biochemical effect of celastrol on liver and kidney function biomarkers in BALB/c mice

Groups	ALT(U/L)	AST(U/L)	BUN(mmol/L)	CREA(μmol/L)
Control	65.13±12.77	170.93±31.78	6.70±0.87	10±2.45
Celastrol (2 mg/kg)	72.43±12.76	172.33±15.32	7.85±0.20	11.67±1.78

Data are expressed as means ± SEM, n = 6 per group. ALT: alanine aminotransferase; AST: aspartate aminotransferase; BUN: blood urea nitrogen; CREA: creatinine.

### Celastrol inhibits tumor growth by inducing apoptosis and decreasing uridine *in vivo*

**Figure 8:**
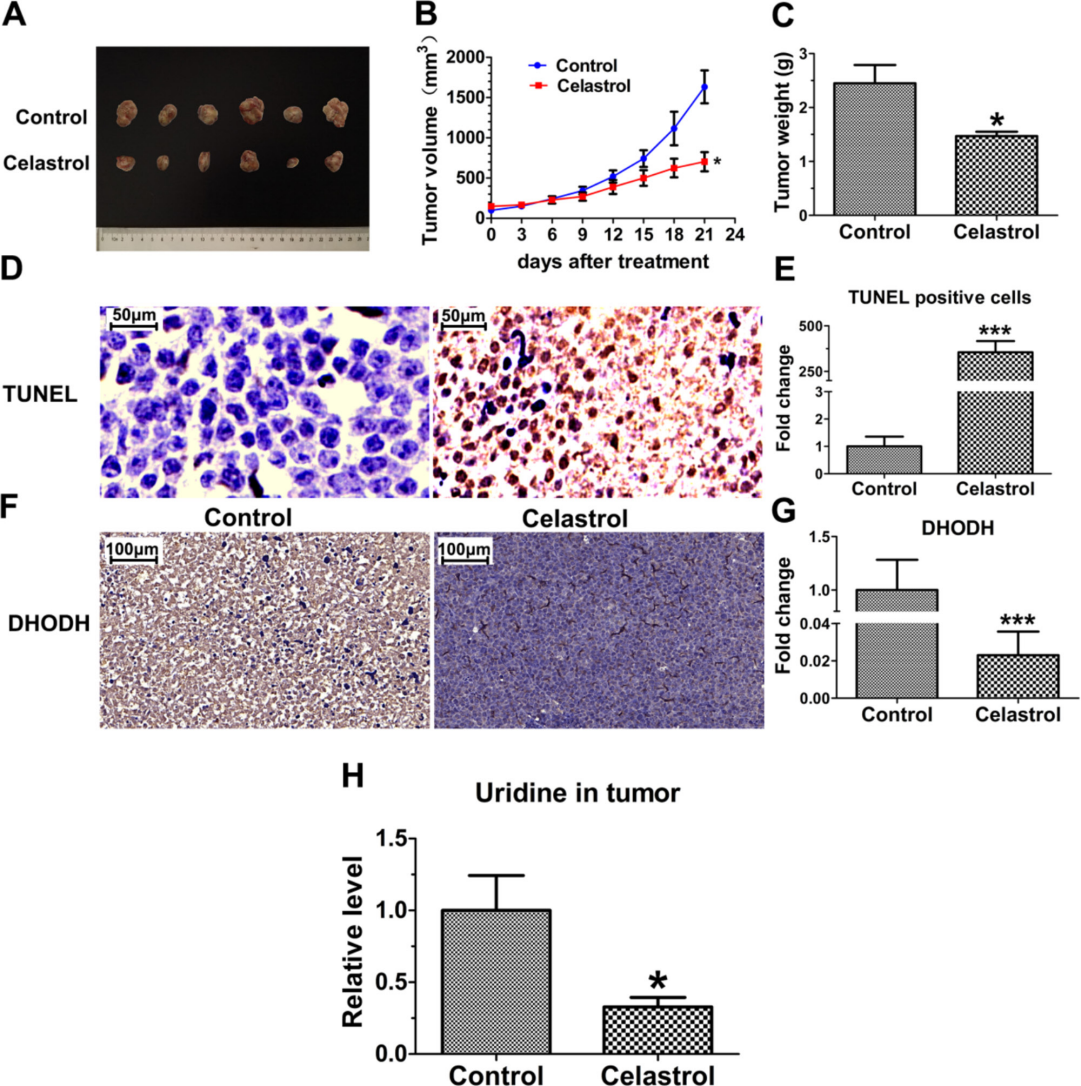
*In vivo* efficacy evaluation of celastrol **A.** Photograph of excised tumors on Day 21 after celastrol treatment. **B.** Tumor growth curves of mice treated with control solvent and celastrol. **C.** The weight of the excised tumor masses from control and celastrol treatment groups. Data expressed as means±SEM (n=6). **D.** Images from TUNEL assay. Scale bar: 50 μm. Magnification, ×40. **E.** Statistical comparison of apoptotic cells in tumor tissues from control and celastrol treatment group. **F.** Immunohistochemistry analysis of DHODH protein level in the tumor tissues with or without celastrol treatment, Scale bar: 100 μm. Magnification, ×20. **G.** The integrated option density of the images from immunohistochemistry analysis was quantified by Image-Pro Plus software. **H.** Comparison of uridine content in tumor tissues from celastrol treated and untreated groups. Asterisks indicate statistical significance (*p < 0.05, ***p < 0.001).

As shown in Figure 8A and 8B, in the model of tumor xenograft nude mice of APL, a significant decrease of tumor volume was observed in celastrol-treated mice compared with control group (753.57±145.41 mm^3^ *vs* 1633.32±289.07 mm^3^). Tumor weight was further confirmed at the end of the experiment after sacrificing mice. As shown in Figure 8C, the tumor weight of celastrol treatment group was significantly reduced compared with that of control (1.47±0.16 g *vs* 2.45±0.68 g). As shown in Supplementary Figure S5, the potent inhibition of celastrol on xenograft tumor can also be supported by the tumor H&E staining, large necrosis areas and a great number of apoptotic cells were observed.

As shown in Figure 8D and 8E, TUNEL assay showed the apoptotic tumor cells were remarkably increased in celastrol-treated group. Meanwhile, consistent with the results *in vitro*, the levels of DHODH protein and uridine in xenograft tumor were decreased significantly (Figure 8F, 8G and 8H), verifying uridine plays a key role in apoptosis of xenograft tumor cells *in vivo*.

## DISCUSSION

**Figure 9:**
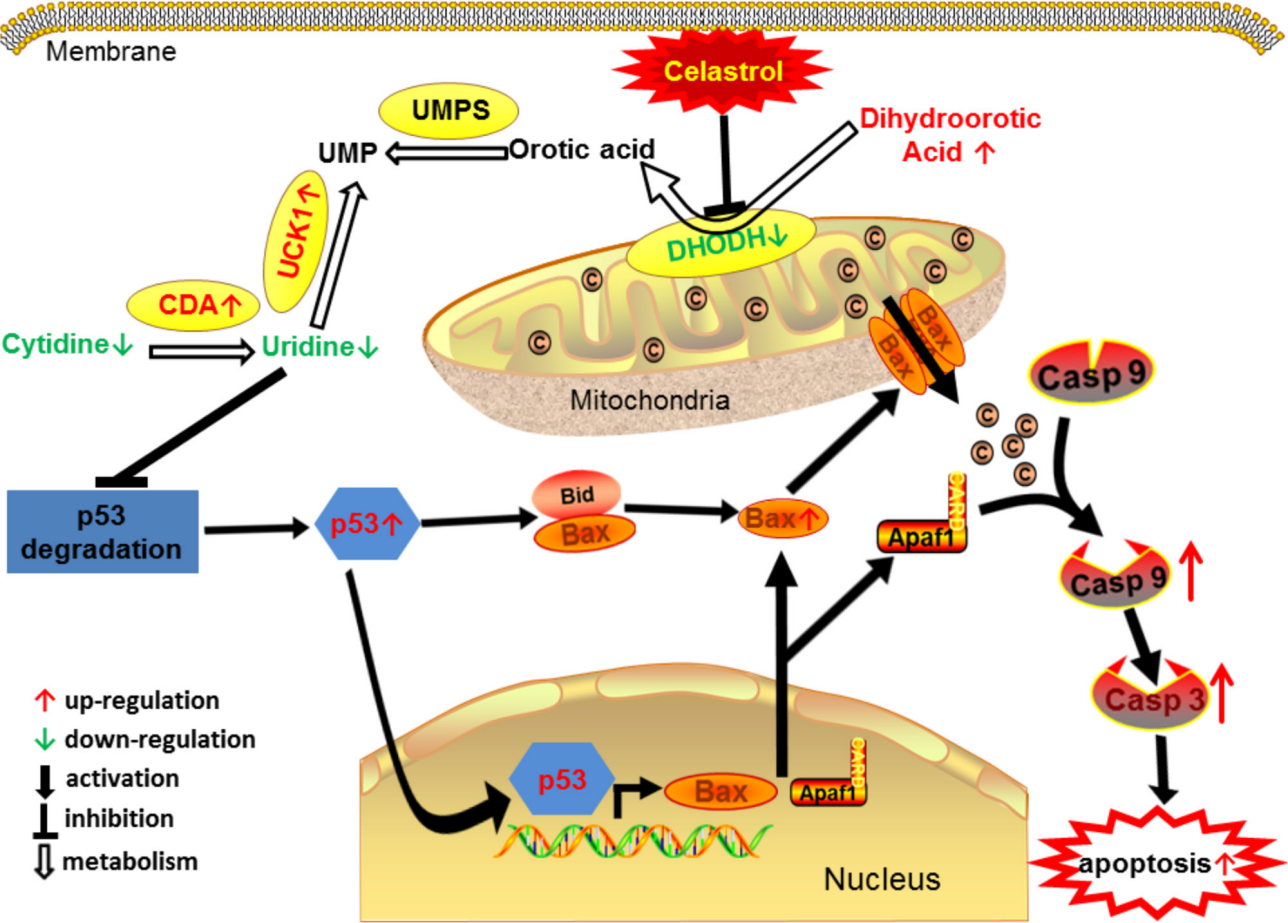
The proposed cascade of events for celastrol induced apoptosis of acute promyelocytic leukemia cells

In this study, from a new perspective, we revealed a novel pathway underlying the inhibitory effect of celastrol on APL cells, namely, DHODH/UCK1/uridine/p53/mitochondrial pathway of apoptosis (Figure 9).

Apoptosis, as a cellular suicide program, plays an essential role as a protective mechanism against tumor cells. Evading apoptosis is a prominent hallmark of cancer [35]. Dysregulation of the apoptotic pathways may not only promote tumorigenesis, but also render the cancer cells resistant to treatment. Therefore, it's very important to control cellular apoptosis strictly for the organisms, especially hematopoietic system as it has a high turnover rate [36]. In this study, we found celastrol could cause apoptosis of APL cells both *in vitro* and *in vivo*, indicating celastrol has anti-leukemia effect through inducing apoptosis.

The downstream of mitochondrial apoptosis pathway has been extensively studied [37]. However, to date, the upstream of this pathway remains to be elucidated. p53 has been considered as a key molecular in cancer progression. It has been reported that p53 can block cell cycle progression and angiogenesis [38, 39]. Recently, the pro-apoptotic effect of p53 in tumor is receiving increasing attention. p53 can trigger apoptosis through mitochondrial pathway by activating Bax [24]. In our study, we found that the pro-apoptotic effect of celastrol on APL cells was through p53-activated mitochondrial pathway. Moreover, as the protein stability of p53 can be affected by uridine [26], based on unbiased metabolomics screening, we found uridine metabolism was a new upstream target involved in p53-activated mitochondrial apoptosis induced by celastrol. These findings also provide us novel insights into the upstream of mitochondrial pathway of apoptosis.

In the present study, uridine deficiency induced by celastrol was caused by the abnormal enhancement of pyrimidine salvage biosynthesis, which resulted from the down-regulation of gene encoding DHODH, the rate-limiting enzyme in *de novo* biosynthesis of pyrimidine. The results indicated that celastrol may be a new inhibitor of DHODH. Interestingly, the inhibitors of DHODH are currently used for the therapy of autoimmune diseases, and celastrol has long been proved to be very effective for this kind of disease [40]. Furthermore, it was reported that the inhibition of DHODH could induce apoptosis [41–43]. Teriflunomide, a well-known specific inhibitor of DHODH, could also induce significant apoptosis of APL cells in our study (Figure 6). The pathway of “celastol/DHODH/uridine/apoptosis” in APL cells revealed in this study extended our current knowledge.

The specificity of drugs on cancer cells is very important for the therapy of cancer. Of note, we found that the APL cells were very sensitive to celastrol. Indeed, celastrol produced significant pro-apoptotic effect on APL cells at concentrations ≤ 1 μM. As previous reports, at this concentration celastrol had no adverse effect on normal cells [13]. Our animal study also confirmed that celastrol had no systemic toxicity on tumor-bearing mice at the pharmacological dose with significant inhibitory effect on tumor growth.

In summary, for the first time, our study reveals “DHODH-induced uridine metabolism disruption/p53/mitochondrial pathway of apoptosis” as a new pathway underlying the effect of celastrol on APL cells, which provides novel insights into the application of celastrol in APL therapy. Our study also shows that metabolomics is a promising tool in pharmacology research.

## MATERIALS AND METHODS

### Cell culture and reagents

Human acute promyelocytic leukemia cell line HL-60 cells, obtained from Cell Bank of Chinese Academy of Sciences (Shanghai, China), were cultured in Iscove's Modified Dulbecco's Medium (IMDM) supplemented with 20% fetal bovine serum, 100 IU/ml penicillin and 100 μg/ml streptomycin. The cells were maintained at 37°C in a humidified incubator with 5% CO_2_.

Purified Celastrol (purity ≥ 98%, CAS No.34158-83-0), uridine (purity ≥ 99%, CAS No.58-96-8), Teriflunomide (purity ≥ 98%, CAS No.163451-81-8) and dimethyl sulfoxide (DMSO) purchased from Sigma-Aldrich (St. Louis, MO, USA). Celastrol was dissolved in DMSO to the concentration of 10 mM. It was then diluted in the culture medium to the final concentration as indicated in every experiment. Uridine was dissolved in PBS at 100 mM.

Cell Counting Kit-8 (CCK-8) was purchased from Dojindo (Kumamoto, Japan).

### Cell proliferation assay

The cell proliferation was monitored by counting viable cells using CCK-8 assay according to the manufacturer's instruction and previous report [44]. Details about the methodology were shown in the Supplementary Information.

### Transmission electron microscope analysis

The cell preparation was performed according to previous study [12]. See Supplementary Information for details of the analysis.

### Flow cytometry analysis

For detection of cell apoptosis, FITC Annexin V Apoptosis Detection Kit I (BD Pharmingen, NJ, USA) was used and performed according to the manufacturer's instruction and more details were according to the previous study [45].

### Multiparametric assay using high content screening assay

Cell-based high-content screening (HCS) multi-parameter cytotoxicity analysis [Thermo Scientific Cellomics®ArrayScan®V^TI^HCS Reader (Pittsburgh, USA)] was used to measure the cell health status of HL-60 cells after celastrol treatment. HL-60 cells were plated in Collagen I-coated 96-well plates (BD Biocoat® Plates, No. 354407) at a density of 2×10^4^/well and incubated for 24 h. After exposure to different concentrations of celastrol (0.125, 0.25 and 0.5 μM) and control solvent for 24 h, the cells were stained using Cellomics® Multiparameter Cytotoxicity 3 Kit (8408102; Cellomics) according to the manufacturer's instruction. More details were according to previous report [46].

### Confocal microscope

The cell preparation was the same as High Content Screening assay. The stained cells were visualized using confocal microscope (Zeiss LSM 700B, Germany).

### RNA isolation and real-time PCR

The mRNA level of apoptosis-related genes (*CASPASE 3*, *CASPASE 9*, *BAX* and *TP53*) and several metabolic enzyme genes (*DHODH*, *UMPS*, *NT5C3A*, *UCK1*, *UCK2* and *CDA*) involved in pyrimidine biosynthesis was detected by real-time PCR. The primer sequences of target genes are listed in Supplementary Table S3, and the melting curves are shown in Supplementary Figure S6.

### Western blot analysis

Whole-cell extracts and western blots were performed according to standard procedures as previously described [47]. Antibodies against cleaved caspase 9, cleaved caspase 3, Bax and p53 were purchased from Cell Signaling Technology (Beverly, MA, USA); anti-DHODH antibody was purchased from Abcam (Cambridge, UK); anti-β-actin antibody and HRP-conjugated secondary antibodies were purchased from Beyotime (Shanghai, China). Detailed methodology is described in the Supplementary Information.

### Metabolomics screening

The cell preparation was performed according to our previous report [46]. In brief, after 24 h of celastrol (0.125, 0.25 and 0.5 μM) and control solvent treatment (n=11, for each group), the cells were harvested and washed with ice-cold PBS for three times. Then 1 ml ice-cold 50% methanol was added to resuspend the cell. After vortex, an equal volume of each sample was pooled together to generate a pooled quality control (Qc) sample. Then all the cells were broken up by ultrasonication for 1 min (power: 60%, pulses: 3/3). Followed by centrifugation (16,000×g, 10 min, 4°C), the supernatant was used for detection. The extracted samples were analyzed with unbiased metabolomics analysis according to our previous study [48]. More details are shown in Supplemental Information.

### Nude mice xenograft tumor assay

Male BALB/c nude mice (aged five weeks and 20-22 g weight) were obtained from NLARSH (Shanghai, China) and housed in a laminar airflow cabinet under specific pathogen-free condition in a 12-h light-dark cycle. After one week acclimation, each mouse was injected subcutaneously with HL-60 cells (5×10^6^ in 100 μl of PBS) under the shoulder as previously described [31–33]. Tumors were allowed to grow until 100 to 200 mm^3^. Then the mice were randomized into two groups (6 mice per group), and were injected intraperitoneally with vehicle control (5% DMSO in 0.9% saline) and celastrol (2 mg/kg/day). The dose of celastrol administration was according to previous reports [11, 12]. Tumor volume and body weights were measured daily. Tumor volume was determined by measuring the tumor in two dimensions with calipers and calculated using the formula: Tumor volume (V) =0.5 ×A×B^2^, where A is the larger diameter and B is the smaller diameter [31]. Mice were sacrificed on day 21 after celastrol treatment. Before sacrifice, mice were maintained under fasting condition for 12 h. Then blood samples were collected. Tumor weight and volume were measured. The organs including heart, liver, spleen, lung, kidneys, brain, testes, and epididymides were removed and weighed. Organ coefficient expressed as tissue weighting factor elsewhere was calculated by dividing weight of individual mouse by the weight of respective organ of that mouse [49]. All animal experiments were performed in accordance with the protocol approved by Nanjing Medical University Institutional Animal Care and Use Committee.

### Serum biomarker analysis

The chronic toxicity of celastrol on liver and kidney was determined by measuring the serum levels of aspartate aminotransferase (AST), alanine aminotransferase (ALT), blood urea nitrogen (BUN) and creatinine (CREA) using blood auto analyzer (Hitachi 7100, Japan) according to previous study [23].

### Hematoxylin and eosin (H&E) analysis and TUNEL assay

The morphological changes of testis and tumor tissues after celastrol treatment were analyzed by H&E staining as described previously [50]. To identify the apoptotic cells in tumor, TUNEL reaction was performed using the ApopTag® Peroxidase In Situ Oligo Ligation (ISOL) Kit (Chemicon International, Inc., Cat #S7200) according to the manufacturer's instruction.

### Immunohistochemistry assay

The protein level of DHODH in tumor tissues was detected by immunohistochemistry assay according to previous study [51].

### Targeted uridine analysis in xenograft tumor in BALB/c nude mice

The concentration of uridine in xenograft tumor was detected using LC-HRMS. The detailed sample preparation and detection can be found in the Supplemental Information.

### Statistical analysis

For metabolomics data, the normalized metabolomics data were imported into SIMCA-P software (Umetrics, Sweden) for multivariate analysis. All the data were UV-scaled and auto log-transformed by the software. Principal Components Analysis (PCA) was applied to find out the metabolic changes between sample groups. The differences of metabolites between treatment group and control were identified by t-test analysis. To improve the statistical robustness, we used a Bonferroni correction for multiple testing with an adjusted p-value threshold of 5×10^−4^. We used the module of “pathway analysis” of metaboanalyst (www.metaboanalyst.ca/) to do the pathway enrichment of metabolomics data.

We used the method of 2^−ΔΔ^Ct to analyze the results of real-time PCR in all experiments. Statistically significant differences between celastrol treatment groups and the control group were determined by one-way ANOVA, followed by Dunnett's multiple comparison tests. Comparisons between two groups were performed by t-test. Values are expressed as means ± standard error (SEM) of the mean for all experiments. All tests of statistical significance were two-sided, and the statistical significance was set at p < 0.05. Analyses and graphs were performed using GraphPad Prism software (San Diego, CA).

## EXTENDED EXPERIMENTAL PROCEDURES


