# Supplementary material for: Metabolomics profiles delineate uridine deficiency contributes to mitochondria-mediated apoptosis induced by celastrol in human acute promyelocytic leukemia cells
Source: Oncotarget. 2016 Jun 25;7(29):46557–72. doi: 10.18632/oncotarget.10286 (PMC5216817; doi:10.18632/oncotarget.10286)
Supplement: Supplementary file 1 [file oncotarget-07-46557-s001.pdf]

Metabolomics profiles delineate uridine deficiency contributes to mitochondria-mediated apoptosis induced by celastrol in human acute promyelocytic leukemia cells

EXTENDED EXPERIMENTAL PROCEDURES

Cell proliferation assay

Briefly, after being seeded in a 96-well microplate at a density of 2×10^5/well, the HL-60 cells were treated with increasing concentrations of celastrol (0.125, 0.25, 0.5, 1, 2, 4 and 8 μM) for 24 and 48 h. Then 10 μl CCK-8 solution was added to each well. After incubation at 37°C for an additional 4 h , the absorbance was measured at 450 nm with the microplate reader (Tecan Infinite 200, Switzerland).

Transmission electron microscope analysis

After celastrol treatment, the HL-60 cells were washed with ice-cold PBS for two times. Then the cells were fixed by 2.5% glutaraldehyde solution overnight at 4°C. The next day the cells were post-fixed with 1% osmium tetroxide. After being dehydrated in increasing concentrations of alcohol, the cell pellets were embedded in epon. Once ultrathin sections were cut, the cells were stained with uranyl acetate and lead citrate. At last, the ultrastructure of the cells was analyzed by transmission electron microscope.

RNA isolation and real-time PCR

Total RNA was isolated using TRIzol reagent (Invitrogen, Carlsbad, CA, USA). The concentration of total RNA was determined using a NanoDrop 2000 (Thermo Fisher Scientific, Wilmington, DE). Reverse transcription and real-time PCR were performed according to the manufacturer's instructions (Takara, Tokyo, Japan). ACTB was used as an endogenous control to determine the expression of target genes by relative quantitation method. Quantification was carried out using ABI7900 Fast Real-Time System (Applied Biosystems, CA, USA) according to the manufacturer's instructions. The specificity of The PCR products was confirmed using melting curve analyses. The $2^{-\Delta\Delta Ct}$ method was used to calculate the relative expression of the target genes. All real-time PCR experiments were repeated at least three times.

Western blot analysis

After celastrol treatment, cells were washed three times in ice-cold PBS and lysed in RIPA Lysis Buffer (containing 50 mM Tris (pH=7.4), 150 mM NaCl, 1% NP-40, 0.5% sodium deoxycholate, 1% SDS, etc.) containing 1mM protease inhibitor PMSF. Cell lysates were centrifugated at 12,000×g for 30 min to harvest supernatants. Protein concentration was quantified using a BCA protein assay kit (Beyotime, China).

Samples containing about 50 μg of total cellular protein were separated via electrophoresis through a SDS–polyacrylamide gel followed by transfer to a PVDF membrane (Millipore, Billerica, MA). Membranes were blocked for 1 h with 5% (w/v) nonfat milk in TBST at room temperature. The blots were incubated with primary rabbit polyclonal antibodies against cleaved caspase 9, cleaved caspase 3, Bax, p53 (Cell Signaling Technology, Beverly, MA, USA), rabbit monoclonal antibody against DHODH(Abcam, Cambridge, UK) and mouse monoclonal antibody against β-actin (Beyotime, China) at 4°C overnight. After being washed with TBST for three times, the membranes were incubated with HRP-conjugated secondary antibody (Beyotime, China) for 1 h at room temperature. The levels of target proteins were detected by ChemiDoc™ MP imaging system (Bio-Rad). Bands were monitored using Immobilon™ Western Chemiluminescent HRP Substrate (Millipore, USA). Western blots were quantified by the Image-Pro Plus software.

Metabolomics analysis

Metabolic profiling was performed on a UPLC Ultimate 3000 system (Dionex, Germering, Germany), coupled to an Orbitrap mass spectrometer (Thermo Fisher Scientific, Bremen, Germany) in both positive and negative mode simultaneously. The chromatographic separation was performed on a 1.9 μm Hypersile Gold C18 column (100 mm×2.1 mm) (Thermo Fisher Scientific), and the column was maintained at 40°C. A multistep gradient had mobile phase A of 0.1% formic acid in ultra-pure water and mobile phase B consisting of acetonitrile (ACN) acidified with 0.1% formic acid. The gradient operated at a flow rate of 0.4 ml/min over a run time of 15 min. The UPLC autosampler temperature was set at 4°C and the injection volume for each sample was 10 μl. All samples were analyzed in a randomized fashion to avoid complications related to the injection order. MS data were collected by the Orbitrap mass spectrometer equipped with a heated electrospray source (HESI). For both positive and

negative mode, the operating parameters were as follows: a spray voltage of 3.5 kV for positive, 2.5 kV for negative, the capillary temperature of 300°C, sheath gas flow of 50 arbitrary units, auxiliary gas flow of 13 arbitrary units, sweep gas of 0 arbitrary units and S-Lens RF level of 60. In the full scan analysis (70 to 1050 amu), the resolution was set at 700,000 with an automatic gain control (AGC) target of 3×10^6 charges. The MS system was calibrated according to the manufacturer's instructions. The chemical identification is based on the retention time and accurate mass with commercial standards. Among the Qc samples, most of the metabolites showed RSDs less than 30%, indicating the metabolomics profiling was reliable [1].

Targeted uridine analysis in xenograft tumor in BALB/c nude mice

50 mg homogenized tumor tissue were mixed with 150 μl ultra-pure water and 600 μl pure methanol. The tissues were ultrasonicated for 5 min (power: 60%, pulses: 3/3), and the supernatant was obtained after centrifugation at 20,000×g for 15 min. After dryness, the residue was reconstituted and then injected into a UPLC Ultimate 3000 system (Dionex), coupled to a Q-Exactive mass spectrometer (Thermo Fisher Scientific) for uridine analysis. The conditions of the mass spectrometer and chromatograph were the same as described in metabolomics analysis. The $[M-H]^-$ ions of uridine at m/z 243.06226 were monitored.

REFERENCE

1. Gika HG, Macpherson E, Theodoridis GA and Wilson ID. Evaluation of the repeatability of ultra-performance liquid chromatography-TOF-MS for global metabolic profiling of human urine samples. J Chromatogr B Analyt Technol Biomed Life Sci. 2008; 871:299-305.

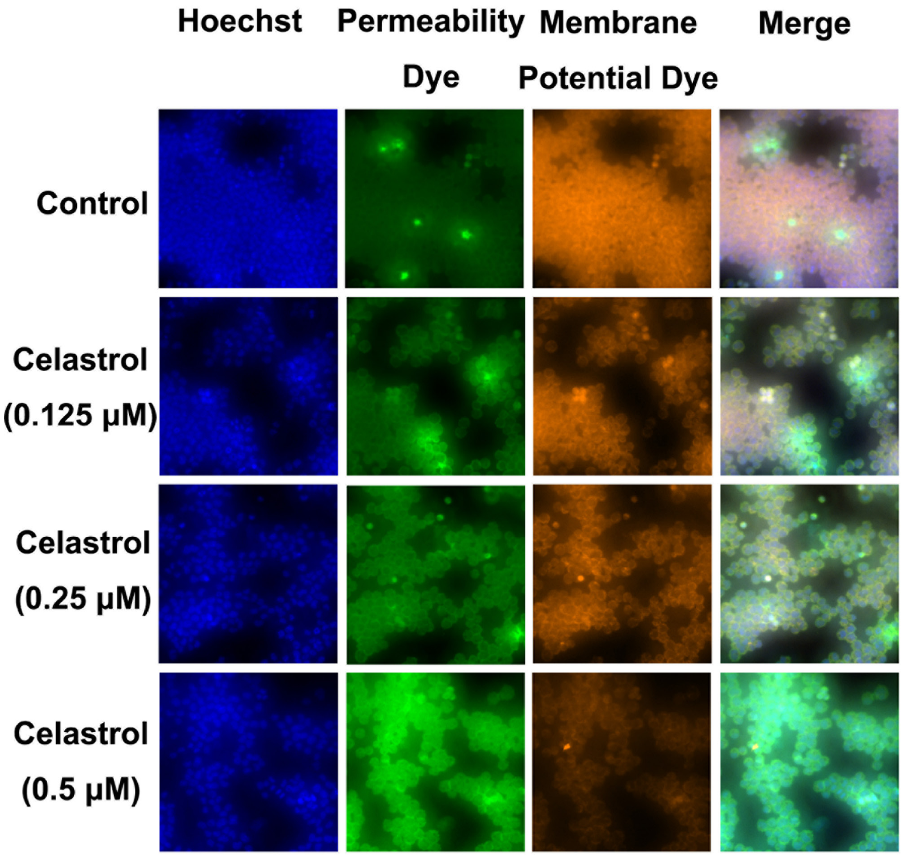

Supplementary Figure S1: Representative images from the High Content Screening after celastrol treatment in HL-60 cells. The nuclei (blue), cell membrane (green) and mitochondria (yellow) can be stained by Hoechst, Permeability Dye, Membrane Potential Dye, respectively. Images were acquired with the ArrayScan HCS Reader with a 20× objective.

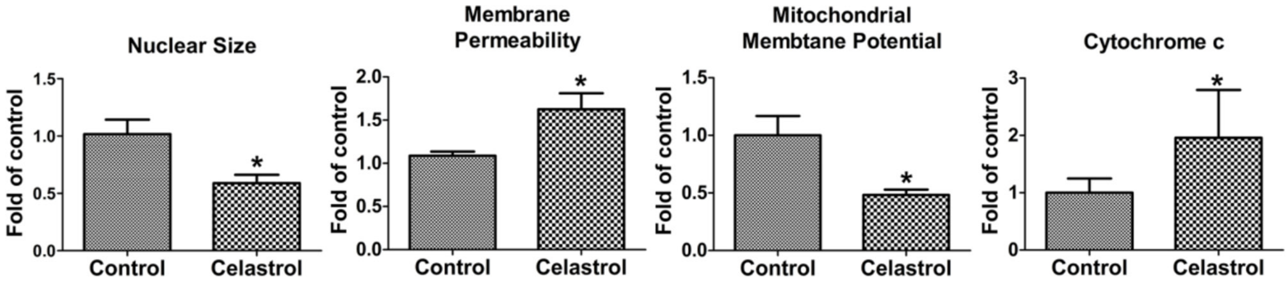

Supplementary Figure S2: Quantitative analysis of images from confocal microscope. The integrated option density (IOD) of each image was quantified by Image-Pro Plus software. Asterisks indicate statistical significance (*p < 0.05).

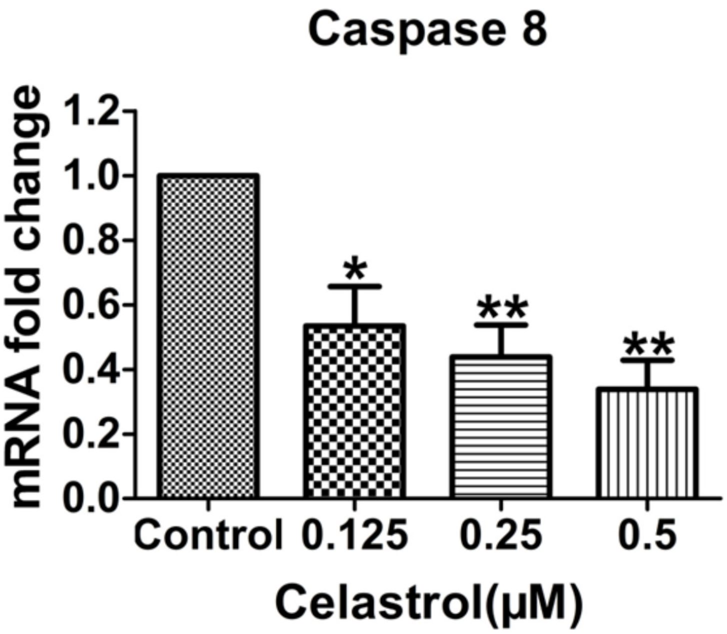

Supplementary Figure S3: The mRNA level of caspase 8. Asterisks indicate statistical significance (*p < 0.05, **p < 0.01, ***p < 0.001).

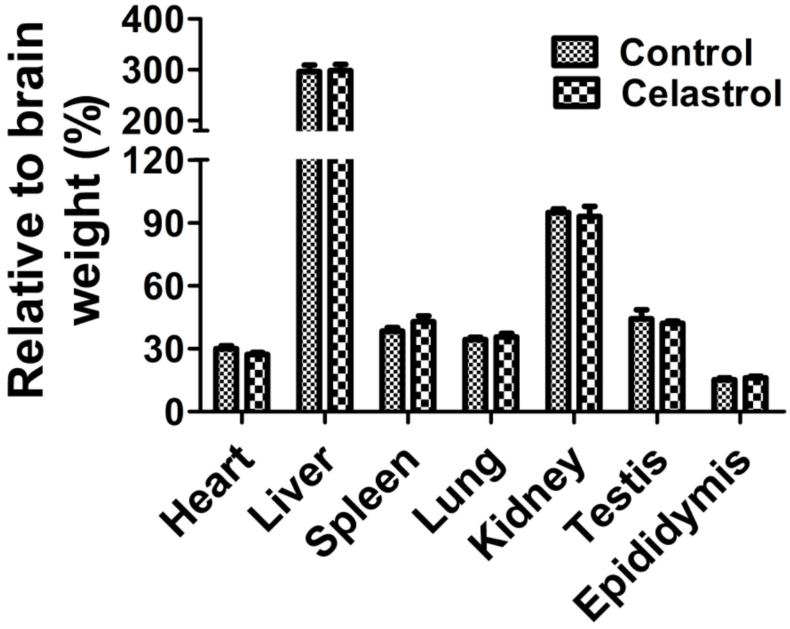

Supplementary Figure S4: The relative weight of heart, liver, spleen, lung, kidneys, testes and epididymides to brain weight.

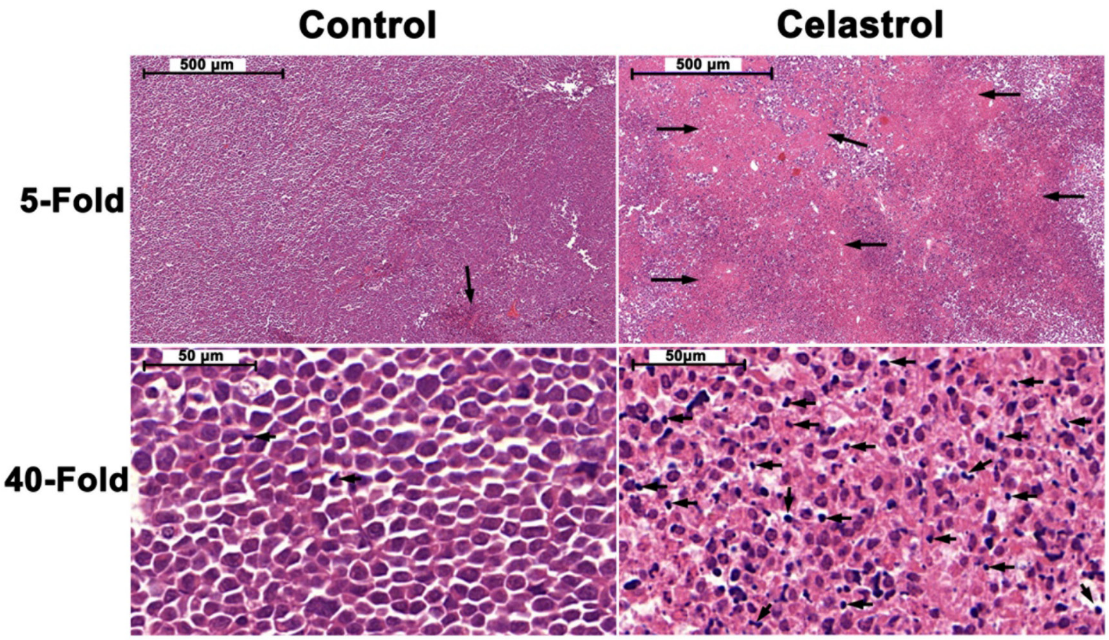

Supplementary Figure S5: Histological (H&E) analysis of tumor tissue sections from different treatment groups. Arrows indicated the necrosis areas in the upper panel or apoptotic cells in the lower panel.

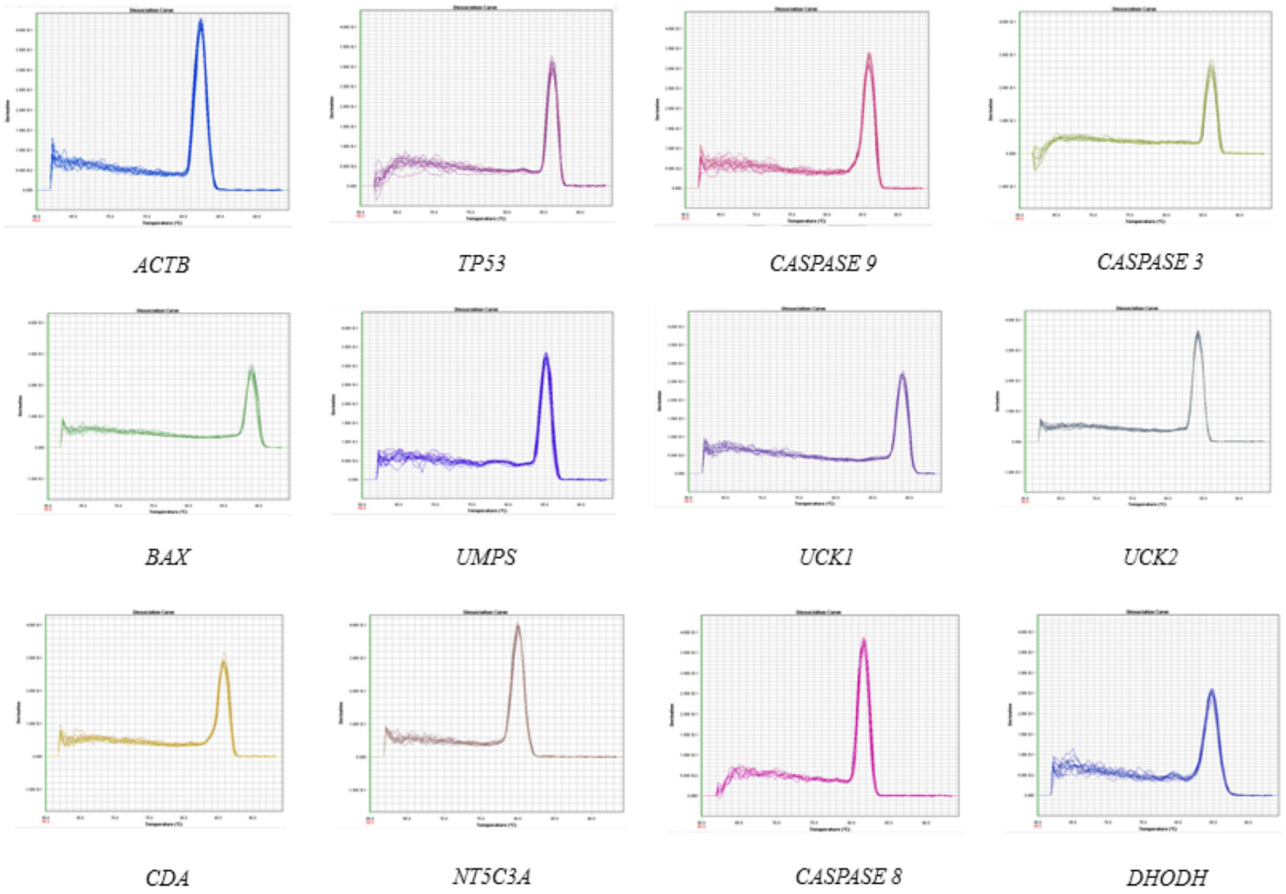

Supplementary Figure S6: The melting curve of real-time PCR analysis.

Supplementary Table S1: List of the metabolites profiled

Compound name	Comparative Analysis							
	Celastrol(0.125µM) *vs* Control		Celastrol(0.25µM) *vs* Control		Celastrol(0.5µM) *vs* Control			
	Fold change	p-value	Fold change	p-value	Fold change	p-value	p<0.0005	p<0.05
Deoxycytidine	1.207	3.51E-01	0.533	2.09E-03	0.024	2.62E-11	#	*
Gluconolactone	0.644	1.51E-03	0.369	7.10E-05	0.104	4.33E-11	#	*
Uridine	0.754	1.62E-02	0.513	2.83E-03	0.100	8.35E-11	#	*
L-Proline	1.482	6.97E-02	0.759	5.63E-02	0.067	9.93E-10	#	*
Argininosuccinic acid	0.714	2.59E-02	1.226	2.78E-01	3.388	3.62E-09	#	*
Hippuric acid	0.944	5.43E-01	0.441	6.49E-05	0.189	1.05E-08	#	*
Cytidine	1.279	2.27E-01	0.775	2.70E-02	0.093	1.19E-08	#	*
Capric acid	0.877	2.36E-01	1.247	3.02E-01	4.209	2.61E-08	#	*
Dodecanoic acid	0.949	4.05E-01	1.488	9.86E-02	4.454	2.66E-08	#	*
Taurine	2.628	5.77E-02	2.534	2.13E-01	0.027	4.52E-08	#	*
3-Methylhistidine	1.288	4.22E-01	1.284	5.12E-01	0.046	4.60E-08	#	*
Glycine	1.385	4.44E-01	0.635	5.37E-02	0.072	4.79E-08	#	*
Ureidopropionic acid	1.254	4.56E-01	1.435	4.18E-01	0.070	5.01E-08	#	*
N-Acetyl-L-methionine	0.496	7.32E-03	0.539	1.22E-02	0.096	6.78E-08	#	*
Spermine	1.485	6.72E-02	1.637	8.60E-02	0.048	7.22E-08	#	*
Xanthosine	0.255	3.41E-05	0.147	3.95E-06	0.015	7.39E-08	#	*
Deoxyinosine	0.756	1.34E-01	0.927	3.31E-02	0.070	1.05E-07	#	*
4-Hydroxyproline	1.148	5.67E-01	1.042	3.37E-01	0.087	1.12E-07	#	*
Biotin	0.294	1.53E-05	0.181	1.03E-06	0.133	1.88E-07	#	*
Riboflavin	0.483	1.91E-03	0.647	2.96E-02	0.097	2.43E-07	#	*
Homocysteine	0.463	1.19E-02	0.496	1.38E-02	0.111	3.29E-07	#	*
D-Glucaric acid	0.432	7.86E-03	0.882	2.98E-01	0.113	4.64E-07	#	*
L-Tryptophan	0.762	7.65E-02	0.650	2.74E-02	0.309	1.85E-06	#	*
L-Acetylcarnitine	1.186	3.07E-01	1.044	8.01E-01	0.169	3.47E-06	#	*
L-Cysteine	1.306	5.88E-01	1.576	3.18E-01	0.053	3.93E-06	#	*
cis-Aconitic acid	0.714	1.03E-01	0.153	1.31E-05	0.123	4.62E-06	#	*
Citric acid	0.757	1.52E-01	0.287	1.78E-04	0.129	6.04E-06	#	*
L-Palmitoylcarnitine	0.541	8.03E-03	0.609	2.65E-02	0.240	7.65E-06	#	*
L-Aspartyl-L-phenylalanine	0.605	1.18E-02	0.555	2.75E-03	0.377	1.35E-05	#	*
L-Histidine	1.580	8.16E-02	1.207	5.32E-01	0.270	1.63E-05	#	*
Gamma-Linolenic acid	1.056	4.34E-01	1.487	2.53E-01	2.559	1.94E-05	#	*
Pantothenol	1.285	1.23E-01	1.053	5.44E-01	7.466	2.56E-05	#	*

(*Continued*)

Compound name	Comparative Analysis							
	Celastrol(0.125µM) *vs* Control		Celastrol(0.25µM) vs Control		Celastrol(0.5µM) *vs* Control			
	Fold change	p-value	Fold change	p-value	Fold change	p-value	p<0.0005	p<0.05
Norvaline	1.120	3.40E-01	1.218	4.01E-01	0.253	3.58E-05	#	*
N-Acetylneuraminic acid	0.544	1.88E-01	1.864	4.84E-01	0.400	2.66E-04	#	*
Eicosapentaenoic acid	0.983	6.34E-01	2.114	1.16E-02	2.093	2.70E-04	#	*
Inosine	0.765	1.23E-01	0.452	3.76E-03	0.425	2.83E-04	#	*
1-Palmitoyl-Palmitoyl	0.604	4.08E-02	1.007	8.14E-01	2.325	3.01E-04	#	*
L-Leucine	0.819	1.96E-01	0.524	6.89E-03	0.394	3.75E-04	#	*
Fumaric acid	0.716	3.23E-01	0.644	2.10E-01	0.104	4.47E-04	#	*
Taurodeoxycholic acid	1.443	3.61E-01	2.004	7.04E-02	3.567	6.29E-04		*
Inosinic acid	2.166	1.91E-02	5.885	2.10E-02	0.214	7.22E-04		*
Pyroglutamic acid	1.226	1.14E-01	0.408	6.18E-04	0.376	9.09E-04		*
L-Lysine	1.261	3.47E-01	2.256	1.87E-02	0.339	1.52E-03		*
Glycerophosphocholine	1.691	1.95E-01	2.477	3.92E-02	0.603	1.73E-03		*
Pyridoxal 5′-phosphate	1.001	8.78E-01	1.902	1.04E-01	0.480	3.48E-03		*
Sorbitol	1.038	8.29E-01	1.409	3.75E-02	2.695	3.70E-03		*
N-Acetyl-L-tyrosine	0.427	1.83E-05	0.755	5.79E-02	0.466	3.73E-03		*
Glucosamine 6-phosphate	1.982	7.53E-02	3.045	9.14E-04	0.469	4.02E-03		*
Guanine	6.257	8.96E-02	3.975	4.75E-02	1.291	4.05E-03		*
Dodecanedioic acid	0.763	6.16E-01	1.991	2.86E-01	1.644	5.21E-03		*
Farnesol	0.889	6.85E-01	1.567	3.99E-02	0.390	5.70E-03		*
Linoleic acid	0.822	7.43E-01	2.250	1.27E-01	1.617	6.85E-03		*
L-Malic acid	0.880	2.58E-01	0.591	6.05E-02	0.352	8.48E-03		*
L-Dihydroorotic acid	0.595	3.74E-01	1.796	1.70E-01	1.945	1.07E-02		*
Allantoin	0.902	8.30E-01	1.025	9.68E-01	0.236	1.09E-02		*
Cortisol	0.323	3.40E-04	0.939	2.16E-01	0.498	1.34E-02		*
Myristic acid	0.748	4.52E-01	2.777	1.28E-01	1.351	1.40E-02		*
Taurocholic Acid	0.795	2.91E-01	2.828	7.40E-02	1.690	1.49E-02		*
Cholesterol	0.225	5.29E-02	0.754	4.80E-01	0.206	1.53E-02		*
2-Hydroxypalmitic Acid	0.622	5.89E-02	0.858	5.52E-01	1.389	1.59E-02		*
Creatinine	0.621	3.04E-01	1.271	6.08E-01	0.470	1.83E-02		*
Arachidonic acid	0.742	3.03E-01	3.443	9.90E-03	3.177	1.89E-02		*
L-Serine	1.562	4.96E-02	1.062	7.49E-01	0.569	2.41E-02		*

(*Continued*)

Compound name	Comparative Analysis							
	Celastrol(0.125µM) *vs* Control		Celastrol(0.25µM) *vs* Control		Celastrol(0.5µM) *vs* Control			
	Fold change	p-value	Fold change	p-value	Fold change	p-value	p<0.0005	p<0.05
Docosahexaenoic acid	0.595	2.45E-01	2.326	1.37E-02	1.990	3.09E-02		*
Valeric acid	0.710	3.72E-01	1.848	2.72E-01	2.016	3.56E-02		*
Rhamnose	0.533	3.81E-01	0.643	5.22E-01	1.334	3.79E-02		*
N-Acetylglutamic acid	0.779	3.53E-01	1.867	3.05E-02	0.305	4.49E-02		*
Glucose 6-phosphate	1.873	6.18E-02	5.405	6.58E-03	0.645	5.68E-02		
Dihydroxyacetone phosphate	1.518	8.07E-02	3.287	1.67E-02	2.380	5.69E-02		
L-Carnitine	1.540	7.39E-02	2.265	4.58E-02	2.163	6.20E-02		
Agmatine Sulfate	2.018	3.01E-01	2.534	1.06E-01	0.527	6.49E-02		
Phthalic acid	0.656	6.05E-01	2.621	1.88E-01	1.221	6.75E-02		
Glycocholic acid	0.885	7.62E-02	0.638	6.38E-02	2.122	7.42E-02		
Adenine	1.611	3.50E-01	5.657	1.95E-01	1.637	1.00E-01		
Folic acid	0.839	7.00E-01	1.277	6.53E-01	1.283	1.20E-01		
Sebacic acid	0.589	2.71E-01	1.424	2.07E-01	0.278	1.40E-01		
3-Pyridylacetic acid	0.897	1.51E-01	2.996	1.17E-02	1.684	1.54E-01		
3-Methyladenine	0.765	5.32E-01	1.814	2.18E-01	0.549	1.92E-01		
Niacinamide	0.750	1.52E-01	1.295	2.52E-01	0.875	2.39E-01		
Glyceraldehyde	0.704	1.77E-01	3.289	2.63E-02	0.919	3.32E-01		
Orotic acid	0.083	2.70E-01	0.868	3.16E-01	0.165	3.70E-01		
Isocitric acid	0.701	1.56E-01	0.520	1.63E-01	0.727	4.42E-01		
Cholic acid	0.359	1.04E-01	0.985	6.35E-01	0.726	4.90E-01		
Tryptamine	0.367	3.83E-01	0.566	6.18E-01	0.409	4.92E-01		
Octadecanedioic acid	0.704	5.93E-01	2.969	1.14E-01	1.096	5.10E-01		
Histamine	0.337	4.53E-01	1.623	1.11E-01	0.418	5.21E-01		
Oxidized glutathione	1.790	2.23E-02	4.028	6.06E-04	1.054	5.26E-01		
Salicylic acid	0.603	3.84E-01	0.512	2.71E-01	1.154	5.65E-01		
Retinal	0.470	5.32E-01	2.187	1.21E-01	0.541	6.24E-01		
Thyroxine	0.677	5.22E-01	2.308	2.46E-01	1.276	7.32E-01		
Octadecanamide	0.557	3.02E-01	2.083	3.22E-01	0.932	7.48E-01		
L-Cystine	0.872	8.29E-01	3.324	7.20E-02	0.919	8.03E-01		
L-Dopa	0.971	2.19E-01	3.339	2.12E-02	1.075	8.07E-01		
Tetradecanedioic acid	0.698	4.28E-01	1.513	4.74E-01	0.962	8.14E-01		
3-Phenylbutyric Acid	0.700	5.99E-01	1.619	5.15E-01	1.076	8.28E-01		

Supplementary Table S2: Metabolic pathway analysis

Pathways	Total	Hits	p-value	Impact
Aminoacyl-tRNA biosynthesis	75	6	8.07E-04	0.000
Nitrogen metabolism	39	4	2.65E-03	0.000
Citrate cycle (TCA cycle)	20	3	3.19E-03	0.135
Arginine and proline metabolism	77	5	5.75E-03	0.189
Pantothenate and CoA biosynthesis	27	3	7.59E-03	0.038
beta-Alanine metabolism	28	3	8.41E-03	0.045
Pyrimidine metabolism	60	4	1.25E-02	0.056
Glutathione metabolism	38	3	1.95E-02	0.035
Glycine, serine and threonine metabolism	48	3	3.60E-02	0.188
Taurine and hypotaurine metabolism	20	2	3.67E-02	0.331
Purine metabolism	92	4	5.04E-02	0.006
Thiamine metabolism	24	2	5.13E-02	0.000
Alanine, aspartate and glutamate metabolism	24	2	5.13E-02	0.003
Histidine metabolism	44	2	1.46E-01	0.145
Phenylalanine metabolism	45	2	1.51E-01	0.032
Biotin metabolism	11	1	1.57E-01	0.203
Primary bile acid biosynthesis	47	2	1.62E-01	0.016
Fatty acid biosynthesis	49	2	1.73E-01	0.000
Glyoxylate and dicarboxylate metabolism	50	2	1.78E-01	0.006
Linoleic acid metabolism	15	1	2.08E-01	0.000
Cyanoamino acid metabolism	16	1	2.20E-01	0.000
Sulfur metabolism	18	1	2.44E-01	0.033
Caffeine metabolism	21	1	2.79E-01	0.000
Riboflavin metabolism	21	1	2.79E-01	0.145

(*Continued*)

Pathways	Total	Hits	p-value	Impact
Phenylalanine, tyrosine and tryptophan biosynthesis	27	1	3.43E-01	0.000
Valine, leucine and isoleucine biosynthesis	27	1	3.43E-01	0.013
Pentose phosphate pathway	32	1	3.93E-01	0.043
Methane metabolism	34	1	4.12E-01	0.000
Butanoate metabolism	40	1	4.65E-01	0.018
Valine, leucine and isoleucine degradation	40	1	4.65E-01	0.022
Nicotinate and nicotinamide metabolism	44	1	4.97E-01	0.000
Ascorbate and aldarate metabolism	45	1	5.05E-01	0.076
Lysine degradation	47	1	5.21E-01	0.000
Fatty acid metabolism	50	1	5.43E-01	0.000
Cysteine and methionine metabolism	56	1	5.84E-01	0.127
Tyrosine metabolism	76	1	6.98E-01	0.000
Tryptophan metabolism	79	1	7.12E-01	0.109
Porphyrin and chlorophyll metabolism	104	1	8.07E-01	0.000

Supplementary Table S3: Oligonucleotide primers used for real-time PCR analysis

Gene	Primer sequences
CASPASE 3	Forward: 5′- GCTATTGTGAGGCGGTTGT-3′<br>Reverse: 5′-GCAGGGCTCGCTAACTC-3′
CASPASE 9	Forward: 5′-CGAACTAACAGGCAAGCA-3′<br>Reverse: 5′-GCACCGACATCACCAAA-3′
CASPASE 8	Forward: 5′-CAGCAAAGGGGAGGAGTT-3′<br>Reverse: 5′-CTTCAAAGGTCGTGGTCAAA-3′
TP53	Forward:5′-GCGTGTGGAGTATTTGGATGAC-3′<br>Reverse: 5′-AGTGTGATGATGGTGAGGATGG-3′
BAX	Forward: 5′-TGCTTCAGGGTTTCATCCA-3′<br>Reverse: 5′-GACACTCGCTCAGCTTCTTG-3′
DHODH	Forward:5′-GGAGATGAGCGTTTCTATGC-3′<br>Reverse: 5′-TGAAGCGAACAGCCAGTC-3′
CDA	Forward: 5′-TGCTGAACGGACCGCTAT-3′<br>Reverse: 5′-AGGGCTGTGAGACATCTTTATG-3′
UCK1	Forward: 5′-TCTGGCAAACGGTCACAT-3′<br>Reverse: 5′-CTCGCTGCTAACACTCCC-3′
UCK2	Forward: 5′-CTTCTACCGTGTCCTTACC-3′<br>Reverse: 5′-TCTCGCTGATGTCCCTTA-3′
UMPS	Forward: 5′-GTCTCAATCGTCAGGTAAGG-3′<br>Reverse: 5′-CAGGTAAACAAGGACAGCAT-3′
NT5C3A	Forward: 5′-GCTCCAACAACATAGCATCC-3′<br>Reverse: 5′-TTCCTCAAGGCACCATCAT-3′
ACTB	Forward: 5′-GCCGAGGACTTTGATTGC-3′<br>Reverse: 5′-GTGGGGTGGCTTTTAGGA-3′
